# Understanding antibody-dependent enhancement in dengue: Are afucosylated IgG1s a concern?

**DOI:** 10.1371/journal.ppat.1011223

**Published:** 2023-03-30

**Authors:** Andrew Teo, Hao Dong Tan, Thomas Loy, Po Ying Chia, Caroline Lin Lin Chua

**Affiliations:** 1 Lee Kong Chian School of Medicine, Nanyang Technological University, Singapore, Singapore; 2 National Centre for Infectious Diseases, Singapore, Singapore; 3 Department of Medicine, The Doherty Institute, University of Melbourne, Melbourne, Australia; 4 School of Biosciences, Faculty of Health and Medicine Sciences, Taylor’s University, Subang Jaya, Malaysia; 5 A*STAR Infectious Diseases Labs, Agency for Science, Technology and Research (A*STAR), Singapore, Singapore; 6 Department of Infectious Diseases, Tan Tock Seng Hospital, Singapore, Singapore; Mount Sinai School of Medicine, UNITED STATES

Dengue is a mosquito-borne infection caused by dengue virus (DENV) of the Flaviviridae family. There are four distinct serotypes, DENV-1,-2,-3,-4, which cause outbreaks globally. Infection is often self-resolving, and lifelong immunity against the infecting serotype can be achieved after exposure. However, in some individuals, homologous infection may still result in symptomatic dengue [1]. There is usually limited protection against heterotypic infections by three other DENV serotypes, as cross-protection is short-lived [2]. After this window of “protection”, subsequent infection with a different serotype may increase the risk of developing severe dengue [3,4]. Antibody-dependent enhancement (ADE) in dengue, a process mainly mediated by immunoglobulin G (IgG), is believed to be one of the major underlying mechanisms leading to increased severity in secondary DENV infection. ADE was shown to enhance viral entry into immune cells via their Fcγ receptors (FcγR), which promotes viral replication, leading to increased viremia and pro-inflammatory responses. These contribute to disease pathologies including vascular hyperpermeability, a common cause of severe dengue [5]. Although this pathological link was first reported about six decades ago, the inherent molecular mechanisms are still not fully understood. Here, we discuss the current model of ADE in dengue and provide new perspective on the possible roles of afucosylated IgG1s in ADE-mediated severe dengue.

## Viral and host factors in influencing ADE in dengue

**Fig 1:**
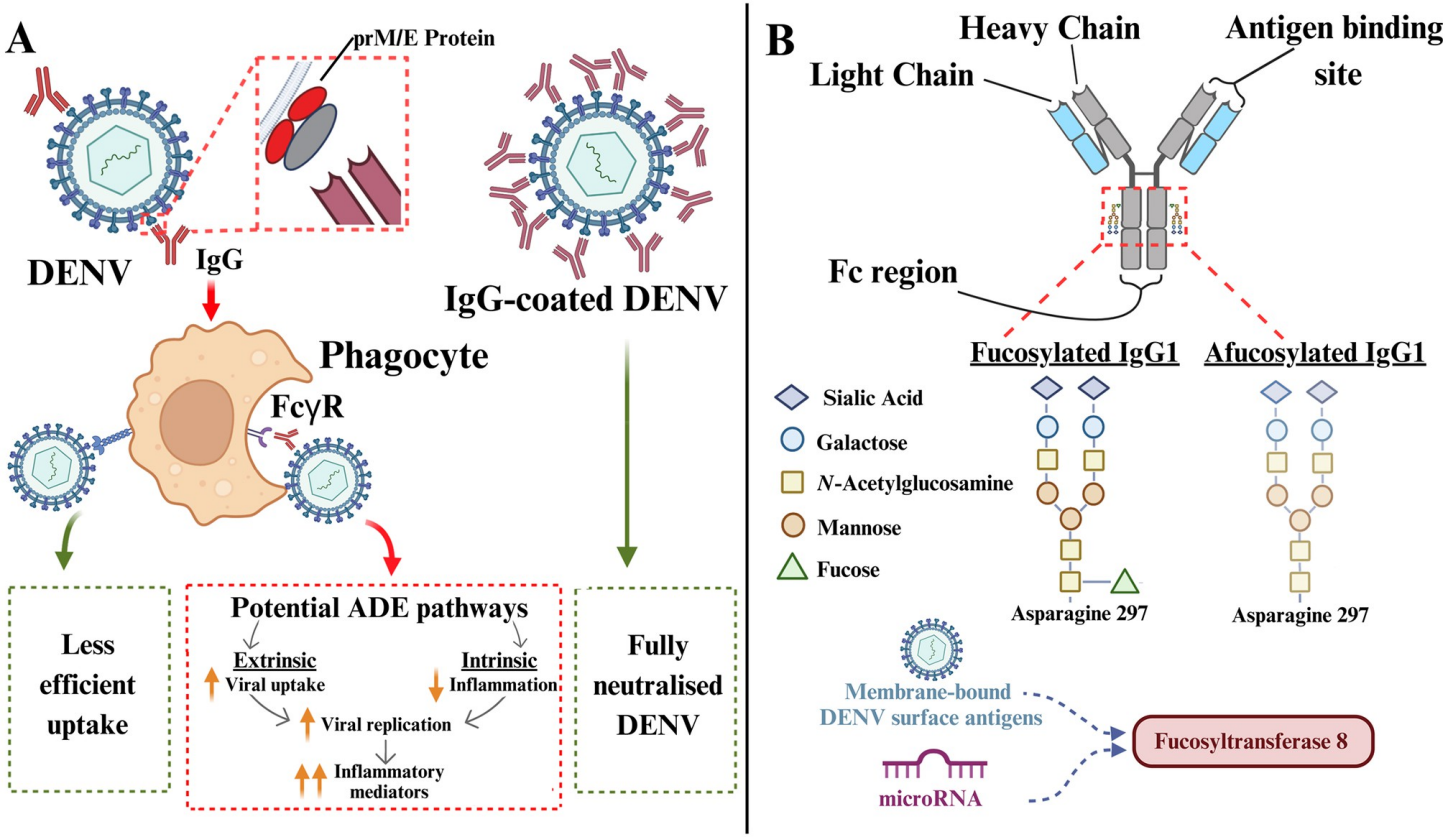
Schematic representation of IgG antibodies and the interaction with DENV. (**A**) Anti-DENV IgGs can bind to DENV antigens including PrM and E proteins, which promotes interaction with FcγRs, such as FcγRI (CD64), FcγRII (CD32), and FcγRIII (CD16), expressed by phagocytes. When these antibodies are insufficient to neutralise the DENV, this may then result in enhanced immature virion uptake into the phagocytes (extrinsic pathway). In the phagocytes, the suppression of a pro-inflammatory response and induction of a Th2-type immune response can further enhance viral replication (intrinsic pathway), and subsequently cause excessive cytokine production. Together, this process is termed as antibody-dependent enhancement. On the other hand, in the absence of IgG, DENV is taken into the phagocytes less efficiently through canonical receptor-mediated endocytosis and is unlikely to contribute to ADE. When neutralising anti-DENV IgGs are present at high levels, DENV is fully neutralised and severe pathology can be prevented. (**B**) IgG is often depicted as a “Y-shaped” molecule consisting of two identical light chains and two identical heavy chains. The antigen-binding site recognises specific antigens, while the Fc region can engage with FcγR expressed by immune cells, leading to activation of effector mechanisms. A posttranslational modification in the Fc region results in the catalysation of a fucose moiety at the asparagine 297 site via FUT8. Afucosylated IgGs lack the fucose structure and have increased binding affinity towards FcγRIII. So far, only afucosylated IgG1s have been reported in dengue patients. The stimuli that trigger IgG1 afucosylation in dengue are not well understood, but DENV surface antigens and microRNAs may play a role in the process by modulating FUT8 expression. ADE, antibody-dependent enhancement; DENV, dengue virus; E, envelope; FcγR, Fcγ receptor; FUT8, fucosyltransferase 8; IgG, immunoglobulin G; PrM, premembrane.

DENV has a positive-sense RNA genome that encodes for seven nonstructural proteins and three structural glycoproteins (the capsid shell, envelope (E) and premembrane (prM) proteins) that are responsible for virus attachment, entry, and maturation [6]. Although all serotypes are closely related, a significant degree of sequence diversity exists [4]. As a result, a subset of IgGs that target viral proteins from one serotype may cross-react with those from other serotypes and exhibit poor neutralising function. For instance, the noninfectious immature virions have a “spiky” appearance, because of higher surface exposure of immunodominant epitopes (pr fragment of the prM proteins and fusion loop in the E proteins) [7]. These epitopes are recognised by low levels of cross-reactive IgGs, promoting entry into FcγR-bearing cells that lead to infectious virus maturation and immune suppression that favours viral replication [4,8,9]. Additionally, decreased neutralising antibody concentrations due to waning immunity may also predispose to ADE. Decay in maternal anti-DENV antibody titres, estimated at <1:20 (determined by serial dilution of plasma via ELISA technique), was associated with increased likelihood of severe dengue during an infant’s primary DENV infection [10]. In another paediatric cohort, high anti-DENV antibody levels, >1:320 titres, protected against symptomatic dengue, whereas individuals with anti-DENV antibody titres between 1:21 and 1:80 were more susceptible to severe dengue [2,3]. A mouse model of dengue was used to delineate ADE pathways, and in pups born to DENV-1 immune dams, older pups (>3 weeks old) were more likely to succumb to vascular leakage and death compared to their younger counterparts (2 weeks old) when challenged with DENV-2. In the same study, sera from older pups displayed lower neutralising activities, and their sera demonstrated ADE-mediated increased viremia in vitro [11]. Similarly, in a nonhuman primate model, passive transfer of monoclonal anti-DENV IgG 1A5 at lower concentration (0.22 to 0.67mg/kg) demonstrated increased viremia titre when challenged with DENV-4 [12]. Overall, these observations reinforce the hypothesis that waning immunity may contribute to ADE-mediated outcomes (Fig 1A).

Given that FcγRs mediate DENV immune complex uptake, their phenotypic and genetic expression on immune cells may also determine the risk of ADE. FcγRs are surface receptors on lymphoid and myeloid cells, with a unique distribution on each cell type. They can be classified as activating or inhibitory receptors, depending on their intracellular domains. In vitro studies reported that FcγRIIa (CD32a) and FcγRI (CD64) mediate ADE in primary monocytes [13], while FcγRIIa and FcγRIIIa (CD16a) were shown to promote ADE in U937 promonocytic cell line [14]. In contrast, interaction between DENV immune complexes and FcγRIIb, an inhibitory FcγR, prevents phagocytosis and reduces the likelihood ADE in monocytes [15]. In populational studies, genetic polymorphism in FcγRIIa results in a single amino acid alteration (arginine instead of histidine at position 131) and is protective against dengue haemorrhagic fever. This is presumably due to increased binding affinity of their FcγRIIa for IgGs, which subsequently accelerates DENV elimination [16,17]. However, due to limited studies, the association between genetic polymorphism in FcγR genes and the risk of ADE is still not well understood. Lastly, immune cell populations and subpopulations are known to express different levels and types of FcγR, which can change during an infection. For example, FcγRIIa expression can be down-regulated in classical monocytes and up-regulated in intermediate and nonclassical monocytes in dengue [18,19]. Whether or not these changes influence the risk of ADE remains to be determined.

## Antigen-specific IgG1s afucosylation and its regulation

In humans, there are four different subclasses of IgGs (IgG1, IgG2, IgG3, and IgG4). Most of the circulating IgGs undergo fucosylation (approximately 94% in adults), a posttranslation modification in the IgG–Fc region where a fucose moiety is attached to the N-linked glycan in the Fc region (Fig 1B) [20]. This creates a steric hindrance and reduces their Fc-FcγRIII binding affinity. In contrast, afucosylated IgGs, which lack the fucose molecule, circulate at low levels under homeostatic condition and have high affinity for FcγRIII. In various infectious diseases, increased levels of antigen-specific afucosylated IgG1s have been reported, and these correlated with different disease severities. For example, afucosylated IgG1s appear to mediate severe disease in enveloped virus infections (COVID-19 and dengue) by promoting immune-mediated pathologies [14,21–23]. While in HIV-1 infected individuals, the presence of antigen-specific afucosylated IgG1 was associated with better control of viral load, suggesting a protective mechanism [24].

The mechanisms and stimuli involved in inducing antigen-specific IgG1 afucosylation in dengue are unknown, but these are likely to be regulated by factors such as exposure to specific DENV antigens, cytokine activities and activation of innate immune signalling receptors. It was proposed that B cell recognition of cell membrane–bound antigens, rather than soluble antigens, in enveloped viral infections, may be one of the stimuli that triggers IgG1 afucosylation. Accordingly, afucosylated antibodies were shown to be specific against E protein of DENV, spike protein of SARS-CoV-2, and envelope glycoprotein of HIV, all of which are antigens associated with host cell membrane [21,23,24]. A recent study hypothesised that the recognition of host cell membrane, in addition to the foreign antigen by B cell, provides a “self” signal to the B cell and may trigger signalling pathways that result in afucosylation; this remains to be proven in future studies [25]. Fucosyltransferase 8 (FUT8) is the enzyme involved in IgG fucosylation. The knockout of *FUT8* gene in cell lines led to increased abundance of afucosylated monoclonal antibodies, while the expression of *FUT8* gene by plasmablast was reported to inversely correlate with IgG1–Fc afucosylation [26,27]. MicroRNAs (miR) may play a role in the regulation of *FUT8* gene, where miR-26a, miR-455-3p, miR-449a, and miR-34a can negatively regulate FUT8 expression in cancer cells (Fig 1B) [28,29]. Both miR-449a and miR-34a were reported to regulate immune responses in DENV infection, but their expression levels in dengue patients and how they may affect IgG1 afucosylation through FUT8 expression in B cells remain elusive [30].

## Afucosylated IgG1s in dengue

While having preexisting anti-DENV antibodies during heterotopic secondary infection predisposes to the risk of severe dengue, only a minority (<5%) progresses to severe outcomes [2,3]. A plausible explanation is that ADE is only mediated by antibodies of particular specificity or type, which leads to different levels of susceptibility in patients. For instance, afucosylated IgG1s have increased binding affinity to FcγRIIIa and FcγRIIIb; this may trigger higher uptake of DENV immune complexes and cause extensive inflammation, which are commonly associated with ADE [14,23]. Afucosylated IgG1s from dengue patients recognise the E proteins, and these antibody levels correlated with increased disease severity [21,22]. Furthermore, in early disease phase, higher afucosylated IgG1 abundance was observed in secondary DENV infection compared to primary infection, and these antibodies were predictive of severe dengue [21]. Having ≥10% of afucosylated anti-E IgG1 was reported to be a significant risk factor for thrombocytopenia and blocking of FcγRIIIa protected against platelet reduction in mice [22]. Similarly, the presence of ≥10% of afucosylated maternal anti-E IgG1s predicted symptomatic primary dengue in infants [14].

## Potential pathways triggered by afucosylated IgG1s in dengue

**Fig 2:**
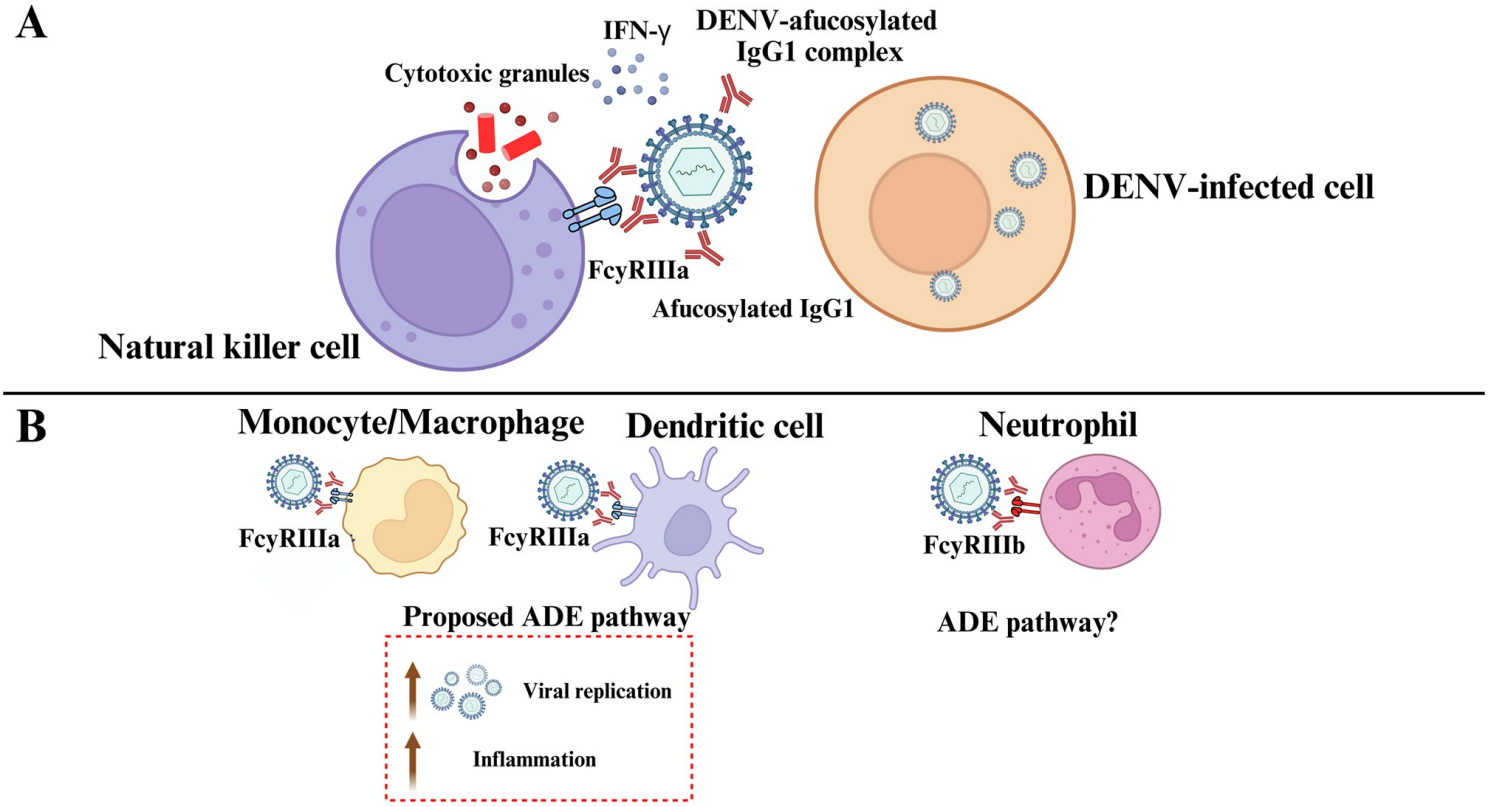
Proposed effector pathways in different immune cells following stimulation with afucosylated IgG1–DENV immune complexes. (**A**) NK cells highly express FcγRIIIa (CD16a) and may recognise afucosylated IgG1–DENV immune complex. This may then promote antibody-dependent cell-mediated cytotoxicity, leading to direct killing of DENV-infected cells and DENV, contributing to protection from symptomatic dengue. (**B**) Afucosylated IgG1s may promote extrinsic ADE in phagocytes, as the absence of fucose molecule greatly enhances the binding of afucosylated IgG1–DENV immune complex to FcγRIIIa expressed by monocytes/macrophages and dendritic cells. This may lead to immature virion uptake, maturation, and replication, leading to increased viremia and inflammation. Neutrophils express FcγRIIIb, and DENV-activated neutrophil can release potent antimicrobial molecules that are associated with severe dengue. However, whether interaction between afucosylated IgG1–DENV immune complex and FcγRIIIb results in ADE and/or release of potent antimicrobial is unknown. Further investigations are needed to provide evidence on afucosylated lgG1s involvement in the proposed pathways above. ADE, antibody-dependent enhancement; DENV, dengue virus; IgG, immunoglobulin G; NK, natural killer.

The downstream pathways triggered by afucosylated IgG1s in dengue and their roles in ADE are not well understood, but these are likely to be dependent on the type of immune cells that they interact with (Fig 2). For example, natural killer (NK) cells highly express FcγRIIIa, and increased binding affinity of afucosylated IgG1s may enhance antibody-dependent cellular cytotoxicity (ADCC). In dengue, ADCC has been correlated with reduced ADE in vitro, and ADCC promoted IFN-γ expression that was associated with protection against symptomatic dengue in humans [31,32] (Fig 2A). Additionally, compared to fucosylated IgGs, afucosylated antibodies can enhance degranulation and killing of tumour cells by NK cells, as well as promoting quicker detachment of the NK cells from target cells, enabling them to attack more cells [33,34]. Together, it is tempting to speculate that patients who can produce high levels of afucosylated IgGs in early primary DENV infection may be able clear the infection more effectively.

In contrast, interaction between afucosylated IgG1s and FcγRIIIa on monocytes/macrophages/dendritic cells may enhance immature virion uptake, thus promoting ADE-mediated viral replication; this is known as the extrinsic ADE pathway (Fig 2B) [9]. Using in vitro monocytic cell line, afucosylated DENV immune complexes engaged FcγRIIIa to increase immunoreceptor tyrosine-based afucosylated IgG motif (ITAM) signalling that enhanced infection [35]. In vivo studies revealed that afucosylated IgG1–FcγRIIIa interacts with splenic macrophages that promoted viral replication and anti-afucosylated IgG1 nanobodies protected against ADE pathogenicity in mice [36,37]. In SARS-CoV-2 infection, the uptake of afucosylated immune complexes by mononuclear cells was shown to trigger pro-inflammatory cytokine production such as TNF, IL-1β, and IL-6 [23]. It is unclear how afucosylated IgG1 immune complexes can affect cytokine production in DENV infection, but the aforementioned pro-inflammatory mediators have been associated with ADE pathology in dengue [9]. On the contrary, DENV immune complexes have also been demonstrated to suppress pro-inflammatory cytokine production in THP-1 monocytic cell line and to subsequently promote viral replication; this is also known as the intrinsic ADE pathway [38]. FcγRIIIb is highly expressed on neutrophils, and activated neutrophils have been associated with severe dengue [5]. Whether afucosylated IgG1s interact with FcγRIIIb to promote ADE or degranulation in neutrophils is unknown. These observations highlight the complexity in delineating the precise ADE-mediated pathology, but it is likely that fucosylated and afucosylated antibodies may induce different inflammatory responses during DENV infection, and further studies to investigate the roles of these antibodies in ADE are warranted.

## Conclusions

In dengue, afucosylated antibodies appear to be correlated with severe outcomes, but the mechanisms by which they contribute to ADE in dengue are unclear. Further studies on the roles of afucosylated IgGs in dengue, and whether their expression differ between naturally exposed and vaccine-induced individuals, are needed to improve our understanding on ADE-mediated pathology in dengue patients.

## References

[1] Waggoner JJ, Balmaseda A, Gresh L, Sahoo MK, Montoya M, Wang C, et al. Homotypic Dengue Virus Reinfections in Nicaraguan Children. J Infect Dis. 2016;214(7):986–993. Epub 2016/03/18 06:00. doi: 10.1093/infdis/jiw099 . 26984144 PMC5021223

[2] Salje H, Cummings DAT, Rodriguez-Barraquer I, Katzelnick LC, Lessler J, Klungthong C, et al. Reconstruction of antibody dynamics and infection histories to evaluate dengue risk. Nature. 2018;557(7707):719–723. Epub 2018/05/26 06:00. doi: 10.1038/s41586-018-0157-4 . 29795354 PMC6064976

[3] Katzelnick LC, Gresh L, Halloran ME, Mercado JC, Kuan G, Gordon A, et al. Antibody-dependent enhancement of severe dengue disease in humans. Science. 2017;358(6365):929–932. Epub 2017/11/04 06:00. doi: 10.1126/science.aan6836 . 29097492 PMC5858873

[4] Dejnirattisai W, Jumnainsong A, Onsirisakul N, Fitton P, Vasanawathana S, Limpitikul W, et al. Cross-reacting antibodies enhance dengue virus infection in humans. Science. 2010;328(5979):745–748. Epub 2010/05/08 06:00. doi: 10.1126/science.1185181 . 20448183 PMC3837288

[5] Teo A, Chua CLL, Chia PY, Yeo TW. Insights into potential causes of vascular hyperpermeability in dengue. PLoS Pathog. 2021;17(12):e1010065. Epub 2021/12/10 06:00. doi: 10.1371/journal.ppat.1010065 . 34882753 PMC8659665

[6] Kuhn RJ, Zhang W, Rossmann MG, Pletnev SV, Corver J, Lenches E, et al. Structure of dengue virus: implications for flavivirus organization, maturation, and fusion. Cell. 2002;108(5):717–725. Epub 2002/03/15 10:00. doi: 10.1016/s0092-8674(02)00660-8 . 11893341 PMC4152842

[7] Kostyuchenko VA, Zhang Q, Tan JL, Ng TS, Lok SM. Immature and mature dengue serotype 1 virus structures provide insight into the maturation process. J Virol. 2013;87(13):7700–7707. Epub 2013/05/03 06:00. doi: 10.1128/JVI.00197-13 . 23637416 PMC3700294

[8] Rodenhuis-Zybert IA, Moesker B, da Silva Voorham JM, van der Ende-Metselaar H, Diamond MS, Wilschut J, et al. A fusion-loop antibody enhances the infectious properties of immature flavivirus particles. J Virol. 2011;85(22):11800–11808. Epub 2011/09/02 06:00. doi: 10.1128/JVI.05237-11 . 21880758 PMC3209313

[9] Narayan R, Tripathi S. Intrinsic ADE: The Dark Side of Antibody Dependent Enhancement During Dengue Infection. Front Cell Infect Microbiol. 2020;10(580096):580096. Epub 2020/10/31 06:00. doi: 10.3389/fcimb.2020.580096 . 33123500 PMC7573563

[10] Simmons CP, Chau TN, Thuy TT, Tuan NM, Hoang DM, Thien NT, et al. Maternal antibody and viral factors in the pathogenesis of dengue virus in infants. J Infect Dis. 2007;196(3):416–424. Epub 2007/06/29 09:00. doi: 10.1086/519170 . 17597456 PMC4333207

[11] Ng JK, Zhang SL, Tan HC, Yan B, Martinez JM, Tan WY, et al. First experimental in vivo model of enhanced dengue disease severity through maternally acquired heterotypic dengue antibodies. PLoS Pathog. 2014;10(4):e1004031. Epub 2014/04/05 06:00. doi: 10.1371/journal.ppat.1004031 . 24699622 PMC3974839

[12] Goncalvez AP, Engle RE, St Claire M, Purcell RH, Lai CJ. Monoclonal antibody-mediated enhancement of dengue virus infection in vitro and in vivo and strategies for prevention. Proc Natl Acad Sci U S A. 2007;104(22):9422–9427. Epub 2007/05/23 09:00. doi: 10.1073/pnas.0703498104 . 17517625 PMC1868655

[13] Boonnak K, Dambach KM, Donofrio GC, Tassaneetrithep B, Marovich MA. Cell type specificity and host genetic polymorphisms influence antibody-dependent enhancement of dengue virus infection. J Virol. 2011;85(4):1671–1683. Epub 2010/12/03 06:00. doi: 10.1128/JVI.00220-10 . 21123382 PMC3028884

[14] Thulin NK, Brewer RC, Sherwood R, Bournazos S, Edwards KG, Ramadoss NS, et al. Maternal Anti-Dengue IgG Fucosylation Predicts Susceptibility to Dengue Disease in Infants. Cell Rep. 2020;31(6):107642. Epub 2020/05/14 06:00. doi: 10.1016/j.celrep.2020.107642 . 32402275 PMC7344335

[15] Chan KR, Zhang SL, Tan HC, Chan YK, Chow A, Lim AP, et al. Ligation of Fc gamma receptor IIB inhibits antibody-dependent enhancement of dengue virus infection. Proc Natl Acad Sci U S A. 2011;108(30):12479–12484. Epub 2011/07/13 06:00. doi: 10.1073/pnas.1106568108 . 21746897 PMC3145677

[16] Mohsin SN, Mahmood S, Amar A, Ghafoor F, Raza SM, Saleem M. Association of FcγRIIa Polymorphism with Clinical Outcome of Dengue Infection: First Insight from Pakistan. Am J Trop Med Hyg. 2015;93(4):691–696. Epub 2015/08/05 06:00. doi: 10.4269/ajtmh.15-0199 . 26240159 PMC4596583

[17] Loke H, Bethell D, Phuong CX, Day N, White N, Farrar J, et al. Susceptibility to dengue hemorrhagic fever in vietnam: evidence of an association with variation in the vitamin d receptor and Fc gamma receptor IIa genes. Am J Trop Med Hyg. 2002;67(1):102–106. Epub 2002/10/05 04:00. doi: 10.4269/ajtmh.2002.67.102 . 12363051

[18] Maheshwari D, Saini K, Singh P, Singla M, Nayak K, Aggarwal C, et al. Contrasting behavior between the three human monocyte subsets in dengue pathophysiology. iScience. 2022;25(6):104384. Epub 2022/05/28 06:00. doi: 10.1016/j.isci.2022.104384 . 35620424 PMC9127603

[19] Aguilar-Briseño JA, Upasani V, Ellen BMT, Moser J, Pauzuolis M, Ruiz-Silva M, et al. TLR2 on blood monocytes senses dengue virus infection and its expression correlates with disease pathogenesis. Nat Commun. 2020;11(1):3177. Epub 2020/06/25 06:00. doi: 10.1038/s41467-020-16849-7 . 32576819 PMC7311456

[20] de Haan N, Reiding KR, Driessen G, van der Burg M, Wuhrer M. Changes in Healthy Human IgG Fc-Glycosylation after Birth and during Early Childhood. J Proteome Res. 2016;15(6):1853–1861. Epub 2016/05/11 06:00. doi: 10.1021/acs.jproteome.6b00038 . 27161864

[21] Bournazos S, Vo HTM, Duong V, Auerswald H, Ly S, Sakuntabhai A, et al. Antibody fucosylation predicts disease severity in secondary dengue infection. Science. 2021;372(6546):1102–1105. Epub 2021/06/05 06:00. doi: 10.1126/science.abc7303 . 34083490 PMC8262508

[22] Wang TT, Sewatanon J, Memoli MJ, Wrammert J, Bournazos S, Bhaumik SK, et al. IgG antibodies to dengue enhanced for FcγRIIIA binding determine disease severity. Science. 2017;355(6323):395–398. Epub 2017/01/28 06:00. doi: 10.1126/science.aai8128 . 28126818 PMC5557095

[23] Larsen MD, de Graaf EL, Sonneveld ME, Plomp HR, Nouta J, Hoepel W, et al. Afucosylated IgG characterizes enveloped viral responses and correlates with COVID-19 severity. Science. 2021;371(6532):23. Epub 2020/12/29 06:00. doi: 10.1126/science.abc8378 . 33361116 PMC7919849

[24] Ackerman ME, Crispin M, Yu X, Baruah K, Boesch AW, Harvey DJ, et al. Natural variation in Fc glycosylation of HIV-specific antibodies impacts antiviral activity. J Clin Invest. 2013;123(5):2183–2192. Epub 2013/04/09 06:00. doi: 10.1172/JCI65708 . 23563315 PMC3637034

[25] Oosterhoff JJ, Larsen MD, van der Schoot CE, Vidarsson G. Afucosylated IgG responses in humans—structural clues to the regulation of humoral immunity. Trends Immunol. 2022;43(10):800–814. Epub 2022/08/26 06:00. doi: 10.1016/j.it.2022.08.001 . 36008258 PMC9395167

[26] Chakraborty S, Gonzalez JC, Sievers BL, Mallajosyula V, Chakraborty S, Dubey M, et al. Early non-neutralizing, afucosylated antibody responses are associated with COVID-19 severity. Sci Transl Med. 2022;14(635):eabm7853. Epub 2022/01/19 06:00. doi: 10.1126/scitranslmed.abm7853 . 35040666 PMC8939764

[27] Malphettes L, Freyvert Y, Chang J, Liu PQ, Chan E, Miller JC, et al. Highly efficient deletion of FUT8 in CHO cell lines using zinc-finger nucleases yields cells that produce completely nonfucosylated antibodies. Biotechnol Bioeng. 2010;106(5):774–783. Epub 2010/06/22 06:00. doi: 10.1002/bit.22751 . 20564614

[28] Cheng L, Gao S, Song X, Dong W, Zhou H, Zhao L, et al. Comprehensive N-glycan profiles of hepatocellular carcinoma reveal association of fucosylation with tumor progression and regulation of FUT8 by microRNAs. Oncotarget. 2016;7(38):61199–61214. Epub 2016/08/18 06:00. doi: 10.18632/oncotarget.11284 . 27533464 PMC5308645

[29] Swierczynski S, Klieser E, Illig R, Alinger-Scharinger B, Kiesslich T, Neureiter D. Histone deacetylation meets miRNA: epigenetics and post-transcriptional regulation in cancer and chronic diseases. Expert Opin Biol Ther. 2015;15(5):651–664. Epub 2015/03/15 06:00. doi: 10.1517/14712598.2015.1025047 . 25766312

[30] Lv J, Zhang Z, Pan L, Zhang Y. MicroRNA-34/449 family and viral infections. Virus Res. 2019;260:1–6. Epub 2018/11/10 06:00. doi: 10.1016/j.virusres.2018.11.001 . 30412711 PMC7114830

[31] Sun P, Williams M, Nagabhushana N, Jani V, Defang G, Morrison BJ. NK Cells Activated through Antibody-Dependent Cell Cytotoxicity and Armed with Degranulation/IFN-γ Production Suppress Antibody-dependent Enhancement of Dengue Viral Infection. Sci Rep. 2019;9(1):1109. Epub 2019/02/03 06:00. doi: 10.1038/s41598-018-36972-2 . 30710094 PMC6358599

[32] Dias AG Jr., Atyeo C, Loos C, Montoya M, Roy V, Bos S, et al. Antibody Fc characteristics and effector functions correlate with protection from symptomatic dengue virus type 3 infection. Sci Transl Med. 2022;14(651):eabm3151. Epub 2022/06/30 06:00. doi: 10.1126/scitranslmed.abm3151 . 35767652 PMC10115655

[33] Karampatzakis A, Brož P, Rey C, Önfelt B, Cruz De Matos GDS, Rycroft D, et al. Antibody Afucosylation Augments CD16-Mediated Serial Killing and IFNγ Secretion by Human Natural Killer Cells. Front Immunol. 2021;12(641521):641521. Epub 2021/04/03 06:00. doi: 10.3389/fimmu.2021.641521 . 33796107 PMC8008054

[34] Liu SD, Chalouni C, Young JC, Junttila TT, Sliwkowski MX, Lowe JB. Afucosylated antibodies increase activation of FcγRIIIa-dependent signaling components to intensify processes promoting ADCC. Cancer Immunol Res. 2015;3(2):173–183. Epub 2014/11/13 06:00. doi: 10.1158/2326-6066.CIR-14-0125 . 25387893

[35] Gonzalez JC, Chakraborty S, Thulin NK, Wang TT. Heterogeneity in IgG-CD16 signaling in infectious disease outcomes. Immunol Rev. 2022;309(1):64–74. Epub 2022/07/06 06:00. doi: 10.1111/imr.13109 . 35781671 PMC9539944

[36] Gupta A, Kao K, Yamin R, Oren DA, Goldgur Y, Du J, et al. Mechanism of glycoform specificity and protection against antibody dependent enhancement by an anti-afucosylated IgG nanobody. bioRxiv. 2023;24(2023). Epub 2023/02/08 06:00. doi: 10.1101/2023.01.23.525277 . 37202422 PMC10195009

[37] Yamin R, Kao KS, MacDonald MR, Cantaert T, Rice CM, Ravetch JV, et al. Human FcγRIIIa activation on splenic macrophages drives the in vivo pathogenesis of dengue disease. bioRxiv. 2022:2022.11.02.514909. doi: 10.1101/2022.11.02.514909

[38] Shukla R, Ramasamy V, Shanmugam RK, Ahuja R, Khanna N. Antibody-Dependent Enhancement: A Challenge for Developing a Safe Dengue Vaccine. Front Cell Infect Microbiol. 2020;10:597. doi: 10.3389/fcimb.2020.572681 33194810 PMC7642463

