# Correction: Understanding antibody-dependent enhancement in dengue: Are afucosylated IgG1s a concern?

**DOI:** 10.1371/journal.ppat.1011736

**Published:** 2023-10-18

**Authors:** Andrew Teo, Hao Dong Tan, Thomas Loy, Po Ying Chia, Caroline Lin Lin Chua

In the Funding statement, one of the funders is listed incorrectly as Ministry of Education (MOE). The correct statement is: CLLC receives support from Ministry of Higher Education (MOHE) Malaysia, under Fundamental Research Grant Scheme: ID FRGS/1/2020/SKK0/TAYLOR/02/1. AT is supported by LKC Medicine Dean’s Postdoctoral Fellowship from Lee Kong Chian School of Medicine, Nanyang Technological University. The funders had no role in study design, data collection and analysis, decision to publish, or preparation of the manuscript.
